# Sparse single-step method for genomic evaluation in pigs

**DOI:** 10.1186/s12711-016-0227-8

**Published:** 2016-06-29

**Authors:** Tage Ostersen, Ole F. Christensen, Per Madsen, Mark Henryon

**Affiliations:** SEGES Pig Research Centre, Axeltorv 3, 1609 Copenhagen V, Denmark; Department of Molecular Biology and Genetics, Center for Quantitative Genetics and Genomics, Aarhus University, P.O. Box 50, 8830 Tjele, Denmark; School of Animal Biology, University of Western Australia, 35 Stirling Highway, Crawley, 6009 WA Australia

## Abstract

**Background:**

In many animal breeding programs, with the increasing number of genotyped animals, estimation of genomic breeding values by the single-step method is becoming limited by excessive computing requirements. A recently proposed algorithm for proven and young animals (APY) is an approximation that reduces computing time drastically by dividing genotyped animals into core and non-core animals, with only computations for core animals being time-consuming. We hypothesized that choosing core animals based on representing all generations, minimizing the relatedness within the core group, or maximizing the number of genotyped offspring, would result in greater accuracies of estimated breeding values (EBV).

**Methods:**

We compared eight different core groups for the three pig breeds DanAvl Duroc, DanAvl Landrace and DanAvl Yorkshire. These eight sparse approximations of the single-step method were evaluated based on correlations of EBV for genotyped animals obtained from the sparse methods with those obtained from the usual version of the single-step method. We used a single-trait model with daily gain as trait.

**Results:**

For core groups that distributed animals across generations, correlations for genotyped animals (from 0.977 to 0.989) were higher than for those that did not distribute core animals across generations (from 0.934 to 0.956). For core groups that maximized the number of genotyped offspring, correlations for genotyped animals (from 0.983 to 0.989) were higher than for other core groups (from 0.934 to 0.981). There was no clear association between low relatedness within the core group and accuracy of approximations.

**Conclusions:**

We found that for core groups that represent all generations and that maximize the number of genotyped offspring, accurate approximations of EBV were obtained. However, we did not find a clear association between accuracy and relatedness within the core group. For the APY method, this is the first study that reports systematic criteria for the creation of core groups that result in more accurate EBV than a similar-sized random core group. Random core groups only ensure across-generation representation. Therefore, we recommend choosing a core group that represents all generations and that maximizes the number of genotyped offspring for single-step genomic evaluation using the APY method.

## Background

To estimate genomic breeding values, the single-step method is the method-of-choice for many animal breeding programs [1–4]. A challenge when using this method is the long computing time when the number of genotyped animals increases [5, 6], which puts a constraint on the estimation of genomic breeding values in most breeding schemes. Misztal et al. [5] proposed a computationally efficient solution to this problem, called the algorithm for proven and young animals (APY). APY computes sparse approximations of the inverse genomic relationship matrix by allocating animals to two groups: core and non-core animals [7]. The algorithm is computationally efficient because it ignores genomic relationships among non-core animals and only requires inversion of the genomic relationship matrix for core animals. Estimated breeding values (EBV) computed with APY can be nearly identical to those computed using the full version of the single-step method, where all genomic relationships are included. The APY approximations become more accurate when the core group size increases [5, 6]. Fragomeni et al. [8] suggested that, when a small number of animals is allocated to the core group to reduce computing time, accurate approximations are obtained when animals in the core group are chosen at random from all genotyped animals. Although other ways of choosing core animals have been proposed [6, 9], no study has reported a formal choice of the core animals that results in more accurate approximations than choosing them at random while keeping the size of the core group constant [5–11]. This is surprising because based on [12], the accuracy of APY is related to how well the animals in the core group represent the independent chromosome segments that are present in the population. This suggests that approximations of EBV obtained with APY could be more accurate by choosing core animals that represent the most independent chromosome segments. We propose three criteria to increase accuracies by choosing animals that represent the most independent chromosome segments: (1) choosing animals from all generations, since new cross-overs occur each generation and thus, new independent chromosome segments are created; (2) minimizing the degree of relatedness within the core group by increasing the number of families in the core group, which should lead to a better representation of independent chromosome segments; and (3) including genotyped parents of genotyped animals in the core group since they represent the independent chromosome segments of their offspring. Based on these assumptions, we hypothesized that choosing core animals based on representing all generations, minimizing the relatedness within the core group, or maximizing the number of genotyped offspring, will increase the accuracy of the resulting EBV. We tested this hypothesis by estimating accuracies of approximations of EBV for daily gain for three Danish pig breeds.

## Methods

We compared eight core groups for the three pig breeds DanAvl Duroc, DanAvl Landrace and DanAvl Yorkshire. EBV for genotyped animals from the sparse single-step methods were correlated with EBV from the usual version of the single-step method. We used a single-trait model on daily gain.

### Sparse single-step

To understand the computing issues of the single-step procedure, first we provide a summary on this method. We used the single-step procedure that was formulated in Christensen et al. [3], in which the inverse pedigree relationship matrix for all animals ${\mathbf{A}}_{\text{full}}^{ - 1}$ is replaced by ${\mathbf{H}}_{{}}^{ - 1}$, where:

$${\mathbf{H}}^{ - 1} = {\mathbf{A}}_{\text{full}}^{ - 1} + \left( {\begin{array}{*{20}c} 0 &\quad 0 \\ 0 &\quad {{\mathbf{G}}_{ }^{ - 1} -{\mathbf{A}}_{22}^{ - 1} } \\ \end{array} } \right)$$

where ${\mathbf{G}} = \left( {1 - {\text{w}}_{\text{a}} } \right){\mathbf{G}}_{\text{a}} + {\text{w}}_{\text{a}} {\mathbf{A}}_{22}$ and ${\mathbf{A}}_{22}^{ - 1}$ is the inverse of the part of the pedigree relationship matrix for genotyped animals, ${\text{w}}_{\text{a}}$ is the weight on the pedigree relationship matrix, and ${\mathbf{G}}_{\text{a}}^{ }$ is the genomic relationship matrix adjusted to the same scale as ${\mathbf{A}}_{22}^{ }$ by the following calculation:

$${\mathbf{G}}_{\text{a}} =\upbeta{\mathbf{G}}_{\text{m}} +\upalpha ,$$

where β and α solve the equations:

$${\text{mean}}\left( {{\text{diag}}\left( {{\mathbf{G}}_{\text{m}} } \right)} \right)\upbeta +\upalpha = {\text{mean}}\left( {{\text{diag}}\left( {{\mathbf{A}}_{22} } \right)} \right),$$

and

$${\text{mean}}\left( {{\mathbf{G}}_{\text{m}} } \right)\upbeta +\upalpha = {\text{mean}}\left( {{\mathbf{A}}_{22} } \right),$$

where mean() represents the mean of the elements and diag() represent the diagonal elements of a matrix.

In the above procedure, matrix ${\mathbf{H}}$ is sub-divided into non-genotyped and genotyped animals and index 2 denotes the genotyped individuals. The genomic relationship matrix ${\mathbf{G}}_{\text{m}}$ is defined as:

$${\mathbf{G}}_{\text{m}} = \left( {{\mathbf{M}} - 2{\mathbf{p}}{\mathbf{1}}^{{\prime }} } \right)\left( {{\mathbf{M}} - 2{\mathbf{p}}{\mathbf{1}}^{{\prime }} } \right)^{{\prime }} / \mathop \sum \limits_{\text{j}}^{ } 2{\text{p}}_{\text{j}} \left( {1 - {\text{p}}_{\text{j}} } \right),$$

where matrix ${\mathbf{M}}$ contains the genotypes coded 0, 1, 2, vector ${\mathbf{p}}$ contains the allele frequencies computed from all genotyped animals, and **1** denotes a vector of ones.

The computationally heavy load of the single-step procedure is partly due to the increasing number of non-zero elements in ${\mathbf{H}}^{ - 1}$, which increases the time necessary for preconditioned conjugate gradient (PCG) iteration, but also partly due to the need to invert ${\mathbf{A}}_{22}$ and ${\mathbf{G}}$. Note that the definition of ${\mathbf{H}}^{ - 1}$ includes both ${\mathbf{G}}_{ }^{ - 1}$ and ${\mathbf{A}}_{22}^{ - 1}$, and if the same elements of these matrices could be zero, an even sparser ${\mathbf{H}}^{ - 1}$ would be achieved.

### Sparse inverse genomic relationship matrix

According to Misztal et al. [5], the inverse genomic relationship matrix, ${\mathbf{G}}_{ }^{ - 1}$, can be approximated by separating the genotyped animals into two groups using the APY algorithm:

$${\mathbf{G}}^{ - 1} = \left[ {\begin{array}{*{20}c} {{\mathbf{G}}_{\text{cc}}^{ - 1} + {\mathbf{G}}_{\text{cc}}^{ - 1} {\mathbf{G}}_{\text{cn}} {\mathbf{D}}_{\text{nn}}^{ - 1} {\mathbf{G}}_{\text{nc}} {\mathbf{G}}_{\text{cc}}^{ - 1} } &\quad { - {\mathbf{G}}_{\text{cc}}^{ - 1} {\mathbf{G}}_{\text{cn}} {\mathbf{D}}_{\text{nn}}^{ - 1} } \\ { - {\mathbf{D}}_{\text{nn}}^{ - 1} {\mathbf{G}}_{\text{nc}} {\mathbf{G}}_{\text{cc}}^{ - 1} } &\quad {{\mathbf{D}}_{\text{nn}}^{ - 1} } \\ \end{array} } \right],$$

where index ${\text{c}}$ denotes animals in the core group, index ${\text{n}}$ denotes animals in the non-core group, and ${\mathbf{D}}_{\text{nn}}$ is a diagonal matrix with dimension equal to number of non-core animals and diagonal elements as:

$${\mathbf{D}}_{{{\text{nn}},{\text{ii}}}} = {\mathbf{G}}_{\text{ii}} - {\mathbf{G}}_{\text{ic}} {\mathbf{G}}_{\text{cc}}^{ - 1} {\mathbf{G}}_{\text{ic}}^{{\prime }} ,$$

where ${\mathbf{G}}_{\text{ic}}$ denotes the ith row of ${\mathbf{G}}_{\text{nc}}$. The APY approximation only requires inversion of the submatrix ${\mathbf{G}}_{\text{cc}}$, which is more time and memory efficient than inversion of the full genomic relationship matrix. Furthermore, the inverse of the genomic relationship matrix approximated with APY is sparse with non-zero blocks among the core animals and between core and non-core animals, but only non-zero diagonal elements among the non-core animals [5].

Although this approach does not require calculation of the full ${\mathbf{G}}$, we did calculate it. For an implementation, it would be sufficient to calculate only ${\mathbf{G}}_{\text{cc}}$, ${\mathbf{G}}_{\text{nc}}$ and ${\mathbf{D}}_{\text{nn}}$, where α and β are estimated based on the core animals only.

### Sparse inverse pedigree relationship matrix for genotyped animals

The inverse pedigree relationship matrix for genotyped animals is also dense, partly because of the numerical inversion. However, APY works very poorly for ${\mathbf{A}}_{22}^{ - 1}$ because the resulting ${\mathbf{H}}^{ - 1}$ is not positive definite (preliminary results not shown). Therefore, ${\mathbf{A}}_{22}^{ - 1}$ needs to be made sparse using e.g., the approach proposed by Faux and Gengler [13] or by Misztal et al. [5].

For pig data, the number of genotyped animals is typically much larger than the number of their non-genotyped ancestors, or at least this will soon be the case with the increasing numbers of genotyped animals. Therefore, the inverse of the pedigree relationship matrix for genotyped animals can be calculated efficiently by absorbing non-genotyped ancestors using the following equation [14]:

$${\mathbf{A}}_{22}^{ - 1} = {\mathbf{A}}^{22} - {\mathbf{A}}^{21} \left( {{\mathbf{A}}^{11} } \right)^{ - 1} {\mathbf{A}}^{12} ,$$

where the inverse of the pedigree relationship matrix for animals in the reduced pedigree for genotyped animals ${\mathbf{A}}_{\text{red}}^{ - 1}$ is sub-divided into:

$${\mathbf{A}}_{\text{red}}^{ - 1} = \left[ {\begin{array}{*{20}c} {{\mathbf{A}}^{11} } & {{\mathbf{A}}^{12} } \\ {{\mathbf{A}}^{21} } & {{\mathbf{A}}^{22} } \\ \end{array} } \right],$$

where superscript 1 denotes non-genotyped animals in the pedigree for genotyped animals, and superscript 2 denotes genotyped animals. This entails that only the usually small part, ${\mathbf{A}}^{11}$, of the inverse pedigree relationship matrix containing non-genotyped animals in the pedigree for genotyped animals needs to be inverted.

According to Misztal et al. [5], sparsity of ${\mathbf{A}}_{22}^{ - 1}$ can be achieved without large consequences by simply setting small elements of ${\mathbf{A}}_{22}^{ - 1}$ equal to zero. A sparse version of ${\mathbf{A}}_{22}^{ - 1}$ was achieved here (95 to 99 % sparsity) by setting elements in the range from −0.0001 to 0.0001 equal to zero. We note that, although the resulting sparse version of ${\mathbf{A}}_{22}^{ - 1}$ was not positive definite for the three datasets studied here, the resulting approximation of ${\mathbf{H}}^{ - 1}$ was positive definite in all cases.

### Data and model

We used data on records of daily gain for pigs of the DanAvl Duroc, DanAvl Landrace and DanAvl Yorkshire breeds that were born between 2009 and 2014; their pedigree was traced back to 1996 (see Table 1 for details on the data). A single-trait model for daily gain with variance components from the routine genomic evaluations was used. All animals were phenotyped for daily gain before genotyping and before selection and mating decisions. Further details on the data are in Ostersen et al. [15]. For all genomic evaluation models, a weight ${\text{w}}_{\text{a}}$ of 0.25 was put on the traditional relationships, which is the standard value used in the routine genomic evaluation for this trait (for details see Christensen et al. [3]).

**Table 1:** Overview of data

	DanAvl Duroc	DanAvl Landrace	DanAvl Yorkshire
Number of observations	110,072	227,786	211,311
Number of animals in pedigree	119,930	239,378	220,998
Number of genotyped animals	13,809	21,681	21,634
Number of animals in pedigree for genotyped animals	25,425	28,774	28,318

Pigs born before August 2013 were genotyped with the Illumina PorcineSNP60 Bead chip and pigs born after this date were genotyped with the 8.5 K GGP-Porcine LD Illumina Bead chip. Missing genotypes were imputed using Beagle version 3.3.2 [16]. The following SNP quality controls were applied: SNPs with a call-rate lower than 90 % across all samples genotyped with the 60 K chip were removed; SNPs with a minor allele frequency lower than 0.01 were filtered out; SNPs that deviated strongly from Hardy–Weinberg equilibrium (p < 10^−7^) were excluded; SNPs that were not mapped in the porcine reference genome build 10.2 [17] were also excluded. A total of 33,028, 37,841 and 36,919 SNPs were retained for the Duroc, Landrace and Yorkshire datasets, respectively. An animal’s genotypes were only retained if they had a call frequency higher than 90 % for that animal. Except for quality control and imputation of SNPs, all other data preparations and analyses were run in R [18] and DMU [19].

### Scenarios evaluated

#### NormalG

This scenario was used as reference for all other scenarios, and was the usual single-step procedure, where matrices for genotyped animals were fully inverted and no sparsity was gained.

#### Random10, Random30, Random50

We chose these core groups at random with subset sizes of 10, 30 and 50 % of the genotyped animals. These scenarios were intended to show the effect of across-generation distribution. In addition to Random10, we used Random30 and Random50 to evaluate the number of core animals required for these pig populations. We investigated different random subsets, but the difference in results between two random subsets of the same size was so small (less than 0.001 difference in correlations for all scenarios), that we only report one. This is in agreement with findings from bovine studies [6].

#### Unrelated10

For this scenario, we chose a core group that included 10 % of the genotyped animals that minimized the average degree of relatedness between core animals. The optimization was achieved using a genetic algorithm [20] by a simulated evolution of a set of potential solutions driven by recombination and mutation, which is based on a fitness function that was the average relationship of the core group.

#### Offspring10

In this scenario, 10 % of the genotyped animals were chosen based on their number of genotyped offspring. Thus, animals were ranked according to number of genotyped offspring and the 10 % animals that had the largest number of genotyped offspring were chosen.

#### OffspringRandom10

This was a combination of the Random10 and Offspring10 scenarios. For the youngest genotyped animals (last year of birth), 10 % of the animals were chosen at random. For the oldest genotyped animals (excluding the last year of birth), 10 % of the animals were chosen based on the number of genotyped offspring. Thus, the resulting core group size across old and young animals was 10 % of all genotyped animals.

#### Old10

This core group consisted of the 10 % oldest genotyped animals. This scenario was used for comparison to the other scenarios, since its characteristics were the opposite of those of the Random10 scenario. Hence, the core group represented only the oldest generations.

#### Young10

This core group consisted of the 10 % youngest genotyped animals. This scenario was used for comparison to the other scenarios, since its characteristics were the opposite of those of the Random10, Unrelated10 and Offspring10 scenarios. Hence, none of the animals in the core group had genotyped offspring, and they were more related and represented only one generation.

#### NormalA

In this scenario, we discarded genotypic information completely to act as a baseline scenario.

### Performance criteria

We evaluated each scenario based on four indicators. First, we calculated Pearson correlations of EBV from each scenario with EBV from the usual version of the single-step procedure for genotyped animals. Second, we calculated these correlations for all animals. The main criterion was the correlation of EBV for genotyped animals. Differences between scenarios were assessed using a Hotelling-Williams *t* test. Third, we evaluated each scenario based on the number of PCG iterations, since they are indicators of how numerically well-conditioned the equations are, which influences computation time. The fourth indicator was the sparsity of ${\mathbf{G}}_{ }^{ - 1} - {\mathbf{A}}_{22}^{ - 1}$, which also influences computation time.

## Results

Ignoring genomic information, as in the NormalA scenario, resulted in correlations of EBV with EBV based on the full single-step method for genotyped animals that were on average equal to 0.873 across the three breeds. In the following, results for alternate APY scenarios are compared to those of the full single-step methods.

### Core animals

Correlations between EBV from the full model and EBV from each of the eight core groups that were created for the three pig breeds were significantly different from each other (p < 0.05). The largest correlations were realized by core groups with animals that were distributed across generations and that had many genotyped offspring. For the scenarios that distributed core animals across generations, i.e. Random10 and OffspringRandom10, correlations for genotyped animals in the three breeds ranged from 0.977 to 0.989 (Tables 2, 3, 4, 5, 6, 7). For the scenarios that did not distribute core animals across generations, i.e. Unrelated10, Old10 and Young10, correlations for genotyped animals ranged from 0.934 to 0.956. Likewise, for core groups that maximized the number of genotyped offspring, i.e. Offspring10 and OffspringRandom10, correlations for genotyped animals ranged from 0.983 to 0.989 (Tables 2, 3, 4, 5, 6, 7). Finally, for the scenarios that did not maximize the number of genotyped offspring, i.e. Random10, Unrelated10, Old10 and Young10, correlations for genotyped animals ranged from 0.934 to 0.981 (Tables 2, 3, 4, 5, 6, 7).

**Table 2:** Correlations between EBV from alternate core groups and EBV from the full single-step model for DanAvl Duroc

Scenario	Cor all	Cor genotyped	PCG iterations	Sparsity of $({\mathbf{G}}_{\varvec{ }}^{ - 1} - {\mathbf{A}}_{22}^{ - 1}$)
NormalG^a^	1	1	301	0.0 %
Random10^b^	0.993	0.981	309	76.9 %
Unrelated10^c^	0.968	0.944	412	77.4 %
Offspring10^d^	0.996	0.985	298	78.5 %
OffspringRandom10^e^	0.997	0.989	287	78.1 %
Random30^b^	0.999	0.997	306	46.5 %
Random50^b^	1.000	0.999	284	23.7 %
Old10^f^	0.947	0.939	405	77.7 %
Young10^g^	0.963	0.934	370	76.1 %
NormalA^h^	0.965	0.901	320	–

Correlations were calculated for all animals (Cor all) and genotyped animals (Cor genotyped)

Number of PCG iterations and sparsity of the matrix involved in the single-step formula $({\mathbf{G}}_{ }^{ - 1} - {\mathbf{A}}_{22}^{ - 1}$)

All correlations were significantly different from each other (p < 0.05)

^a^NormalG is the usual single-step procedure without sparse approximations

^b^Random10, Random30, Random50 are the sparse single-step, where a random subset of animals (10, 30, 50 %) were treated as core

^c^Unrelated10 is 10 % animals chosen as core by minimizing the degree of relatedness between core animals

^d^Offspring10 is 10 % animals chosen based on the number of genotyped offspring

^e^OffspringRandom10 is, for old animals (excluding last year of birth) 10 % animals chosen based on the number of genotyped offspring, whereas for young animals (last year of birth) 10 % of the animals were chosen at random

^f^Old10 is the sparse single-step, where the 10 % oldest animals were treated as core

^g^Young10 is the sparse single-step, where the 10 % youngest animals were treated as core

^h^NormalA is where genotypes are ignored completely

**Table 3:** Correlations between EBV from alternate core groups and EBV from the full single-step model for DanAvl Landrace

Scenario	Cor all	Cor genotyped	PCG iterations	Sparsity of $({\mathbf{G}}_{ }^{ - 1} - {\mathbf{A}}_{22}^{ - 1}$)
NormalG^a^	1	1	306	0.0 %
Random10^b^	0.995	0.977	377	80.1 %
Unrelated10^c^	0.982	0.954	492	79.9 %
Offspring10^d^	0.987	0.983	330	80.4 %
OffspringRandom10^e^	0.991	0.984	340	80.3 %
Random30^b^	0.997	0.996	346	48.4 %
Random50^b^	0.996	0.999	312	24.7 %
Old10^f^	0.944	0.936	463	80.1 %
Young10^g^	0.897	0.937	444	79.7 %
NormalA^h^	0.977	0.858	321	–

Correlations were calculated for all animals (Cor all) and genotyped animals (Cor genotyped)

Number of PCG iterations and sparsity of the matrix involved in the single step formula $({\mathbf{G}}_{ }^{ - 1} - {\mathbf{A}}_{22}^{ - 1}$)

All correlations were significantly different from each other (p < 0.05)

^a^NormalG is the usual single-step procedure without sparse approximations

^b^Random10, Random30, Random50 are the sparse single-step, where a random subset of animals (10, 30, 50 %) were treated as core

^c^Unrelated10 is 10 % animals chosen as core by minimizing the degree of relatedness between core animals

^d^Offspring10 is 10 % animals chosen based on the number of genotyped offspring

^e^OffspringRandom10 is, for old animals (excluding last year of birth) 10 % animals chosen based on the number of genotyped offspring, whereas for young animals (last year of birth) 10 % of the animals were chosen at random

^f^Old10 is the sparse single-step, where the 10 % oldest animals were treated as core

^g^Young10 is the sparse single-step, where the 10 % youngest animals were treated as core

^h^NormalA is where genotypes are ignored completely

**Table 4:** Correlations between EBV from alternate core groups and EBV from the full single step model for DanAvl Yorkshire

Scenario	Cor all	Cor genotyped	PCG iterations	Sparsity of $({\mathbf{G}}_{ }^{ - 1} - {\mathbf{A}}_{22}^{ - 1}$)
NormalG^a^	1	1	303	0 %
Random10^b^	0.995	0.978	348	80.1 %
Unrelated10^c^	0.985	0.956	471	80.0 %
Offspring10^d^	0.997	0.984	319	80.4 %
OffspringRandom10^e^	0.997	0.985	325	80.4 %
Random30^b^	0.999	0.996	321	48.4 %
Random50^b^	1.000	0.999	292	24.7 %
Old10^f^	0.967	0.946	442	80.2 %
Young10^g^	0.980	0.943	439	79.8 %
NormalA^h^	0.976	0.858	300	–

Correlations were calculated for all animals (Cor all) and genotyped animals (Cor genotyped)

Number of PCG iterations and sparsity of the matrix involved in the single step formula $({\mathbf{G}}_{ }^{ - 1} - {\mathbf{A}}_{22}^{ - 1}$)

All correlations were significantly different from each other (p < 0.05)

^a^NormalG is the usual single-step procedure without sparse approximations

^b^Random10, Random30, Random50 are the sparse single-step, where a random subset of animals (10, 30, 50 %) were treated as core

^c^Unrelated10 is 10 % animals chosen as core by minimizing the degree of relatedness between core animals

^d^Offspring10 is 10 % animals chosen based on the number of genotyped offspring

^e^OffspringRandom10 is, for old animals (excluding last year of birth) 10 % animals chosen based on the number of genotyped offspring, whereas for young animals (last year of birth) 10 % of the animals were chosen at random

^f^Old10 is the sparse single-step, where the 10 % oldest animals were treated as core

^g^Young10 is the sparse single-step, where the 10 % youngest animals were treated as core

^h^NormalA is where genotypes are ignored completely

**Table 5:** Summary statistics for animals in the core groups for the Duroc breed for alternate scenarios for choice of the core group

Scenario	Mean pedigree relatedness within core	Mean of absolute values of columns of ${\mathbf{A}}_{22}^{ - 1}$ for core	Mean number of genotyped offspring	Part of genotyped animals in core for each birth year				
				2014	2013	2012	2011	<2011
NormalG^a^	0.21	0.0005	1.0	1	1	1	1	1
Random10^b^	0.21	0.0006	1.0	0.10	0.10	0.11	0.10	0.10
Unrelated10^c^	0.15	0.0006	0.7	0	0	0.01	0.05	0.44
Offspring10^d^	0.19	0.0022	9.8	0	0.08	0.17	0.13	0.23
OffspringRandom10^e^	0.21	0.0021	9.4	0.09	0.06	0.13	0.10	0.13
Random30^b^	0.21	0.0005	1.0	0.31	0.29	0.31	0.29	0.30
Random50^b^	0.21	0.0006	1.0	0.50	0.48	0.50	0.50	0.51
Old10^f^	0.15	0.0008	1.6	0	0	0	0	0.48
Young10^g^	0.25	0.0003	0	0.29	0	0	0	0

^a^NormalG is the usual single-step procedure without sparse approximations

^b^Random10, Random30, Random50 are the sparse single-step, where a random subset of animals (10, 30, 50 %) were treated as core

^c^Unrelated10 is 10 % animals chosen as core by minimizing the degree of relatedness between core animals

^d^Offspring10 is 10 % animals chosen based on the number of genotyped offspring

^e^OffspringRandom10 is, for old animals (excluding last year of birth) 10 % animals chosen based on the number of genotyped offspring, whereas for young animals (last year of birth) 10 % of the animals were chosen at random

^f^Old10 is the sparse single-step, where the 10 % oldest animals were treated as core

^g^Young10 is the sparse single-step, where the 10 % youngest animals were treated as core

**Table 6:** Summary statistics for animals in the core groups for the Landrace breed for alternate scenarios for choice of the core group

Scenario	Mean pedigree relatedness within core	Mean of absolute values of columns of ${\mathbf{A}}_{22}^{ - 1}$ for core	Mean number of genotyped offspring	Part of genotyped animals in core for each birth year				
				2014	2013	2012	2011	<2011
NormalG^a^	0.25	0.0004	1.3	1	1	1	1	1
Random10^b^	0.25	0.0004	1.5	0.10	0.10	0.10	0.10	0.09
Unrelated10^c^	0.17	0.0004	1.2	0.01	0.03	0.08	0.17	0.73
Offspring10^d^	0.23	0.0016	12.5	0.01	0.15	0.16	0.11	0.27
OffspringRandom10^e^	0.25	0.0015	11.4	0.09	0.09	0.11	0.08	0.15
Random30^b^	0.25	0.0004	1.4	0.29	0.30	0.31	0.31	0.29
Random50^b^	0.25	0.0004	1.4	0.50	0.50	0.52	0.50	0.48
Old10^f^	0.18	0.0006	2.5	0	0	0	0.17	1
Young10^g^	0.27	0.0002	0	0.21	0	0	0	0

^a^NormalG is the usual single-step procedure without sparse approximations

^b^Random10, Random30, Random50 are the sparse single-step, where a random subset of animals (10, 30, 50 %) were treated as core

^c^Unrelated10 is 10 % animals chosen as core by minimizing the degree of relatedness between core animals

^d^Offspring10 is 10 % animals chosen based on the number of genotyped offspring

^e^OffspringRandom10 is, for old animals (excluding last year of birth) 10 % animals chosen based on the number of genotyped offspring, whereas for young animals (last year of birth) 10 % of the animals were chosen at random

^f^Old10 is the sparse single-step, where the 10 % oldest animals were treated as core

^g^Young10 is the sparse single-step, where the 10 % youngest animals were treated as core

**Table 7:** Summary statistics for animals in the core groups for the Yorkshire breed for alternate scenarios for choice of the core group

Scenario	Mean pedigree relatedness within core	Mean of absolute values of columns of ${\mathbf{A}}_{22}^{ - 1}$ for core	Mean number of genotyped offspring	Part of genotyped animals in core for each birth year				
				2014	2013	2012	2011	<2011
NormalG^a^	0.21	0.0004	1.3	1	1	1	1	1
Random10^b^	0.20	0.0004	1.3	0.10	0.10	0.10	0.11	0.11
Unrelated10^c^	0.12	0.0004	1.5	0	0.02	0.07	0.21	0.84
Offspring10^d^	0.19	0.0016	12.2	0.01	0.15	0.17	0.12	0.27
OffspringRandom10^e^	0.21	0.0014	11.2	0.09	0.08	0.12	0.10	0.14
Random30^b^	0.21	0.0004	1.3	0.30	0.30	0.30	0.32	0.30
Random50^b^	0.21	0.0004	1.2	0.50	0.50	0.50	0.53	0.49
Old10^f^	0.13	0.0006	2.5	0	0	0	0.28	1
Young10^g^	0.24	0.0002	0	0.21	0	0	0	0

^a^NormalG is the usual single-step procedure without sparse approximations

^b^Random10, Random30, Random50 are the sparse single-step, where a random subset of animals (10, 30, 50 %) were treated as core

^c^Unrelated10 is 10 % animals chosen as core by minimizing the degree of relatedness between core animals

^d^Offspring10 is 10 % animals chosen based on the number of genotyped offspring

^e^OffspringRandom10 is, for old animals (excluding last year of birth) 10 % animals chosen based on the number of genotyped offspring, whereas for young animals (last year of birth) 10 % of the animals were chosen at random

^f^Old10 is the sparse single-step, where the 10 % oldest animals were treated as core

^g^Young10 is the sparse single-step, where the 10 % youngest animals were treated as core

Correlations did not increase with decreasing pedigree relatedness between the core animals. The scenario with the lowest average pedigree relationship between core animals, Unrelated10, was not among the most accurate scenarios. However, there was no clear pattern between low relatedness and accuracy of approximations. For the core groups with the lowest average pedigree relatedness between core animals, i.e. Unrelated10 and Old10, correlations for genotyped animals ranged from 0.936 to 0.956 (Tables 2, 3, 4, 5, 6, 7). For the core groups with the highest relatedness between core animals, i.e. Young10, OffspringRandom10 and Random10, correlations for genotyped animals ranged from 0.934 to 0.989.

We observed strong confounding between low relatedness and across-generation distribution, i.e. the Unrelated10 and Old10 scenarios, for which older animals were favored, both showed a poor across-generation distribution compared to Random10 (Tables 5, 6, 7). Similarly, we observed some confounding between low relatedness and number of genotyped offspring, i.e. Old10 and Unrelated10 had some genotyped offspring, as opposed to Young10 (Tables 5, 6, 7), which made it difficult to determine whether the differences in correlations between these scenarios were due to differences in relatedness or in number of genotyped offspring.

### Iterations and sparsity

The scenarios that performed well in terms of accuracy also tended to perform well in terms of number of iterations needed for convergence and sparsity (Tables 2, 3, 4). For instance, for the Offspring10 and OffspringRandom10 scenarios, for which correlations were highest, 6 to 12 % fewer PCG iterations were required compared to the Random10 scenario. Furthermore, for the Offspring10 and OffspringRandom10 scenarios, sparsity was improved by 0.2 to 1.6 percentage units compared to Random10 (Tables 2, 3, 4).

### Proportion of genotyped animals in the core group

To obtain correlations for genotyped animals higher than 99.5 %, the size of the core groups had to be greater than 30 % for all three breeds when the animals were chosen randomly. Choosing a size of 50 % instead of 30 % increased correlations for genotyped animals from an average of 0.996 to an average of 0.999 (Tables 2, 3, 4).

## Discussion

Choosing core animals from all generations in the genotyped population (Random10) and maximizing the number of genotyped offspring (OffspringRandom10), resulted in accurate approximations of EBV based on the APY method. This, however, only partly supports our hypothesis, since we could not find a clear association between relatedness within the core group (Unrelated10) and accuracy of the APY approximation. The increases in accuracy that we found for the three pig breeds when using OffspringRandom10 are useful when estimating breeding values for breeding schemes with time constraints for breeding value estimation. Using the APY approximation, accurate breeding values can be achieved with less computing time and without burdening the breeding program with additional costs. This is the first study to report criteria that realize more accurate EBV than a random core group of similar size. It deviates from the indication of Fragomeni et al. [6], who proposed that the choice of animals for the core group was mostly arbitrary. We found, however, that the choice of core animals is important for the accuracy of APY. This was best highlighted by the Unrelated10, Old10 and Young10 scenarios, for which the least related, old or young animals were chosen. In these scenarios, approximations of EBV were less accurate, which indicates that the choice of core animals is not arbitrary. Therefore, when using APY to reduce computing time, we recommend choosing core animals from all generations and that have the largest number of genotyped offspring.

As we mentioned, one possible reason for the more accurate approximations realized with the OffspringRandom10 scenario (for which the core group included animals from across generations and animals with many genotyped offspring), was that the core group for this scenario represented a greater proportion of the independent chromosome segments from the genotyped animals than the other scenarios. Including animals from all generations presumably increased the number of independent chromosome segments in the core group because each generation is expected to generate new cross-overs and, hence, new independent chromosome segments. This means that the core group should ensure an equal representation of genotyped animals in each generation. However, choosing animals with many genotyped offspring further increases the number of independent chromosome segments represented in the core group because parents represent the independent chromosome segments of their offspring. This reasoning can also be demonstrated mathematically, since animals that have many genotyped offspring represent the columns of the sparse version of ${\mathbf{A}}_{22}^{ - 1}$ with most non-zero elements. Approximately, the same animals have the largest sums of absolute deviations from zero for elements in ${\mathbf{A}}_{22}^{ - 1}$, ${\mathbf{G}}_{ }^{ - 1}$ and ${\mathbf{G}}_{ }^{ - 1} - {\mathbf{A}}_{22}^{ - 1}$. As a result, choosing animals for the core group with large numbers of offspring causes less numerical change in ${\mathbf{H}}_{ }^{ - 1}$. Therefore, the optimal core group represents the largest number of independent chromosome segments and the least numerical change in ${\mathbf{H}}_{ }^{ - 1}$.

Reducing relatedness within the core group did not improve the accuracy of the APY approximation. This is because reducing relatedness favored old animals without many genotyped offspring, which counteracts the two criteria that were found to increase the accuracy of the approximation, i.e. maximizing the number of genotyped offspring and the distribution across generations. Thus, for maximizing accuracy, it is sufficient to maximize the number of genotyped offspring while ensuring across-generation representation and reducing relatedness of the core group is less important.

For core groups with animals that were chosen at random, approximations were nearly as accurate as in the OffspringRandom10 and Offspring10 scenarios. The reason for this good performance is presumably that the randomly selected core group ensures across-generation representation but it does not ensure that animals with many genotyped offspring are chosen. This good performance of random core groups is probably the reason why no other studies detected a core group that performed better than a similar-sized random group of animals—although this was not the goal of any of these studies [5–11]. Lourenco et al. [9, 11] also evaluated a core group based on genotyped offspring, but they did not compare it with a similar-sized random core group, which makes it difficult to evaluate if such a core group performed better. Because of the convincing results of a randomly chosen core group, Fragomeni et al. [6] stated that the choice of animals for the core group is mostly arbitrary. However, our results indicate that it is not completely arbitrary, since it was easy to choose animals in such a way that accuracy of the EBV decreased considerably. For instance, with the Unrelated10, Old10 and Young10 scenarios, accuracies of EBV were lower and computation times were longer, compared to the Random10 scenario, presumably because of the poor across-generation representation. This shows that it is easy to choose animals for the core group that will lead to a decrease in the accuracy of EBV compared to a randomly selected core group. Although these conclusions are based on rather small core group sizes of 10 %, we also tested these conclusions on larger core group sizes of 30 %, which resulted in the same conclusions (unpublished results).Therefore, it is clear that the choice of animals in the core group is not arbitrary.

We set out to find a core group that performed better than a random core group, and we found an improved core group for all generations except the last generation. In the last generation, which has no genotyped offspring, we examined many ways of choosing the core group (results not shown). We examined a core group that was chosen based on, the smallest number of parents in the core group of the older generations, smallest number of genotyped parents, largest sums of absolute deviations from zero in ${\mathbf{A}}_{22}^{ - 1}$, and different family limitations such as a maximum of one core animal per litter. These alternative ways of choosing the last generation all performed well, but none performed better than a random sample. We noted that there was little variation for the last generation both in terms of number of non-zero elements in ${\mathbf{A}}_{22}^{ - 1}$, and sums of absolute deviations from zero in ${\mathbf{A}}_{22}^{ - 1}$. This indicates that there is little to be gained by a systematic choice of young animals. Therefore, we believe that there is very little to be gained in terms of a good representation of independent chromosome segments and little numerical change in ${\mathbf{H}}_{ }^{ - 1}$ by choosing the last generation non-randomly.

We found that treating 30 % of the genotyped animals as the core animals, corresponding to 4500 to 6500 animals, was sufficient to achieve correlations higher than 99 % for genotyped animals. It is not clear from our study whether the number of core animals needed to obtain an accurate approximation is a constant or a percentage of the number of genotyped animals. Results (not shown) on subsets of our data indicated that the minimum percentage of animals required in the core group decreased as the number of genotyped animals increased. When the total number of genotyped animals was reduced to one third, the minimum percentage required as core animals increased to about 50 % of the genotyped animals, corresponding to 2500 to 3500 animals. This is presumably because the small subset of genotyped animals did not include as many independent chromosome segments, and therefore required fewer core animals to represent them. Whether the optimal size of the core group becomes a constant for larger numbers of genotyped animals, as argued by Fragomeni et al. [6] in cattle, remains unclear. We recommend a performance surveillance of the sparse single-step model at larger data sizes, since this issue cannot be investigated based on our current size of data. Regardless of whether the optimal core size is a constant or a decreasing percentage, the sparsity of ${\mathbf{G}}_{ }^{ - 1}$ measured as a percentage will increase as the number of genotyped animals increases, and this will increase the computational gains from sparse single-step methods as the number of genotyped animals increases. Therefore, the sparse single-step method can be applied to pig breeding—especially when there are more than 20,000 genotyped animals.

The number of core animals needed to obtain an accurate approximation is expected to be a function of the number of independent chromosome segments, and thus of the degree of linkage disequilibrium (LD). The smaller number of core animals needed for the Duroc compared to the Landrace and Yorkshire breeds, could be explained by the somewhat higher LD over short distances in the Duroc breed [21]. This can also explain why the number of animals needed in the core group was smaller for the Danish pig breeds compared to Holstein cattle, since Danish pig breeds have a higher degree of LD than Holstein cattle [21, 22]. Another explanation for the smaller core group size for Duroc could be that fewer SNPs are available for this breed, which has been shown to affect the dimensionality of ${\mathbf{G}}$ [23]. Thus, differences in the number of core animals needed between the Danish pig breeds and with Holstein cattle can be explained by the level of LD in the population and the number of SNPs genotyped.

## Conclusions

We found that for core groups representing all generations and maximizing the number of genotyped offspring, APY approximations of EBV were accurate. However, we did not find a clear association between accuracy and relatedness within the core group. This is the first study to report systematic criteria that realize more accurate EBV than a similar-sized random core group, which only ensures across-generation representation. Therefore, we recommend choosing a core group that represents all generations and maximizes the number of genotyped offspring.
